# RE: Chemopreventive Agents to Reduce Mammographic Breast Density in Premenopausal Women: A Systematic Review of Clinical Trials

**DOI:** 10.1093/jncics/pkab051

**Published:** 2021-06-14

**Authors:** John L Hopper, Tuong L Nguyen, Shuai Li

**Affiliations:** Centre for Epidemiology and Biostatistics, Melbourne School of Population and Global Health, University of Melbourne, Parkville, Victoria, Australia

Salazar and colleagues (1) conducted a review of clinical trials of the effect of chemoprevention on mammographic breast density (MBD) and found that “a limited number of chemoprevention agents have been shown to reduce MBD.” The authors note that higher MBD is only “associated with” an increased risk of breast cancer but raise the prospect that “MBD could serve as a surrogate marker of breast cancer development … for preventive interventions.”

But this could occur only if MBD is in fact causal for breast cancer. The authors said that “it is worth noting that breast cancer arises from other biological pathways unrelated to, or completely independent of MBD” (1), and their use of the word *other* implies MBD truly is a “biological pathway.”

It is incontrovertible that MBD causes breast cancers to be missed at mammographic screening because the mammographically dense (white and bright) regions can mask existing tumors from detection. Therefore, higher MBD would cause an increase in the incidence of interval breast cancers (cancers diagnosed following a negative screen during the time interval before the next regular screen). That is, MBD impedes the ability of mammographic screening to identify cancers earlier.

In a screening population, reducing MBD would decrease the incidence of interval cancers and increase the incidence of screen-detected cancers. Given the latter is substantially higher than the former, this would result in an increase in a woman’s overall risk of being diagnosed with breast cancer.

The authors concluded that trials are needed to identify chemoprevention agents that can “reduce MBD” (1). They considered that this “has great potential to open up new opportunities for breast cancer prevention.” No, it won’t. Reducing MBD is not a way to prevent breast cancer—it has the opposite effect, as explained in the next paragraph.

So, how is it that MBD is associated with an increased risk of screen-detected breast cancer? There are other aspects of a mammogram that are associated with increased risk of screen-detected breast cancer, based on brightness and texture, and these are positively correlated with MBD (2-5). When each of these new measures was fitted with MBD, the MBD association attenuated toward the null. When the new measures were combined and fitted with MBD, the MBD association became marginally negative (6). Therefore, it is possible that the positive association of MBD with screen-detected breast cancer is because of confounding with other mammographic features that are causal (7). When this confounding is taken into account, the true causal effect of MBD is a decrease in breast cancer incidence.

In summary, finding ways to decrease MBD has the potential to prevent women from dying from breast cancer. But doing so will naturally lead to an increase in breast cancer incidence overall. If conclusions are to be made about whether MBD is a “biological pathway” or a “surrogate marker for preventive interventions,” trials need to study breast cancer as the outcome, not just MBD. They should also study other aspects of mammograms associated with risk.

## Funding

JLH is supported by a NHMRC Senior Principal Research Fellowship. TLN is supported by Cancer Council Victoria. SL is supported by a Victorian Cancer Agency Early Career Research Fellowship (ECRF19020).

## Notes

**Role of the funders:** The funders had no role in the study design, data collection, and analysis; decision to publish; or preparation of the manuscript.

**Disclosures:** The authors have no conflict of interest to declare.

**Author contributions:** All authors contributed to the conceptualization and writing, both original draft preparation and review and editing.

## Data Availability

Not applicable.
